# Recombinant methioninase combined with doxorubicin (DOX) regresses a DOX-resistant synovial sarcoma in a patient-derived orthotopic xenograft (PDOX) mouse model

**DOI:** 10.18632/oncotarget.24996

**Published:** 2018-04-10

**Authors:** Kentaro Igarashi, Kei Kawaguchi, Shukuan Li, Qinghong Han, Yuying Tan, Emily Gainor, Tasuku Kiyuna, Kentaro Miyake, Masuyo Miyake, Takashi Higuchi, Hiromichi Oshiro, Arun S. Singh, Mark A. Eckardt, Scott D. Nelson, Tara A. Russell, Sarah M. Dry, Yunfeng Li, Norio Yamamoto, Katsuhiro Hayashi, Hiroaki Kimura, Shinji Miwa, Hiroyuki Tsuchiya, Fritz C. Eilber, Robert M. Hoffman

**Affiliations:** ^1^ AntiCancer, Inc., San Diego, California, USA; ^2^ Department of Surgery, University of California, San Diego, California, USA; ^3^ Department of Orthopaedic Surgery, Kanazawa University, Kanazawa, Japan; ^4^ Division of Hematology-Oncology, University of California, Los Angeles, CA, USA; ^5^ Department of Surgery, Yale School of Medicine, New Haven, Connecticut, USA; ^6^ Department of Pathology, University of California, Los Angeles, CA, USA; ^7^ Division of Surgical Oncology, University of California, Los Angeles, CA, USA

**Keywords:** synovial sarcoma, patient-derived orthotopic xenograft, PDOX, recombinant methioninase, doxorubicin

## Abstract

Synovial sarcoma (SS) is a recalcitrant subgroup of soft tissue sarcoma (STS). A tumor from a patient with high grade SS from a lower extremity was grown orthotopically in the right biceps femoris muscle of nude mice to establish a patient-derived orthotopic xenograft (PDOX) mouse model. The PDOX mice were randomized into the following groups when tumor volume reached approximately 100 mm^3^: G1, control without treatment; G2, doxorubicin (DOX) (3 mg/kg, intraperitoneal [i.p.] injection, weekly, for 2 weeks; G3, rMETase (100 unit/mouse, i.p., daily, for 2 weeks); G4 DOX (3mg/kg), i.p. weekly, for 2 weeks) combined with rMETase (100 unit/mouse, i.p., daily, for 2 weeks). On day 14 after treatment initiation, all therapies significantly inhibited tumor growth compared to untreated control, except DOX: (DOX: *p* = 0.48; rMETase: *p <* 0.005; DOX combined with rMETase < 0.0001). DOX combined with rMETase was significantly more effective than both DOX alone (*p <* 0.001) and rMETase alone (*p <* 0.05). The relative body weight on day 14 compared with day 0 did not significantly differ between any treatment group or untreated control. The results indicate that r-METase can overcome DOX-resistance in this recalcitrant disease.

## INTRODUCTION

Sarcoma is a group of 50 or more rare recalcitrant cancers [1]. In order to improve the outcome of sarcoma patients, we have developed patient-derived orthotopic xenograft (PDOX) models of the major sarcomas: soft-tissue sarcoma [2–4], follicular dendritic-cell sarcoma [5], Ewing’s sarcoma [6–10], undifferentiated pleomorphic soft-tissue sarcoma [11, 12], osteosarcoma [13–16, 25], rhabdomyosarcoma [17, 18], leiomyosarcoma [19] and undifferentiated spindle-cell sarcoma (USCS) [20, 23, 26]. During the course of finding more efficacious agents for this group of diseases, we have found that our developmental therapeutic, recombinant methioninase (rMETase), is active and can inhibit or arrest tumor growth and in combination with an appropriate chemotherapy drug, can regress the PDOX tumors [7, 21–26].

rMETase effectively reduced tumor growth of a DOX-resistant *FUS-ERG* Ewing’s sarcoma PDOX [6] compared to untreated control. The methionine level both of plasma and supernatants derived from sonicated tumors was lower in the rMETase group [7].

An undifferentiated spindle-cell sarcoma (USCS) PDOX model was treated with rMETase. rMETase inhibited tumor growth, measured by tumor volume, compared to untreated controls and first-line therapy doxorubicin (DOX). Tumor L-methionine levels were reduced after rMETase-treatment [26].

In a subsequent study, we determined the efficacy of rMETase in combination with DOX in a PDOX model of USCS. rMETase in combination with DOX could regress the USCS PDOX model [23].

Synovial sarcoma (SS) is an aggressive subtype that accounts for 10% to 20% of soft tissue sarcoma (STS) in the adolescent and young adult population [27]. Treatment for localized SS consists of a combination of surgery, radiotherapy and, in some cases, chemotherapy. Chemotherapy has a low response rate for metastatic disease [28]. Treatment is usually wide surgical excision with adjuvant or neoadjuvant therapy. In the present study, we determined if rMETase could overcome first-line DOX-resistance in a PDOX model of SS.

## RESULTS AND DISCUSSION

**Figure 1:**
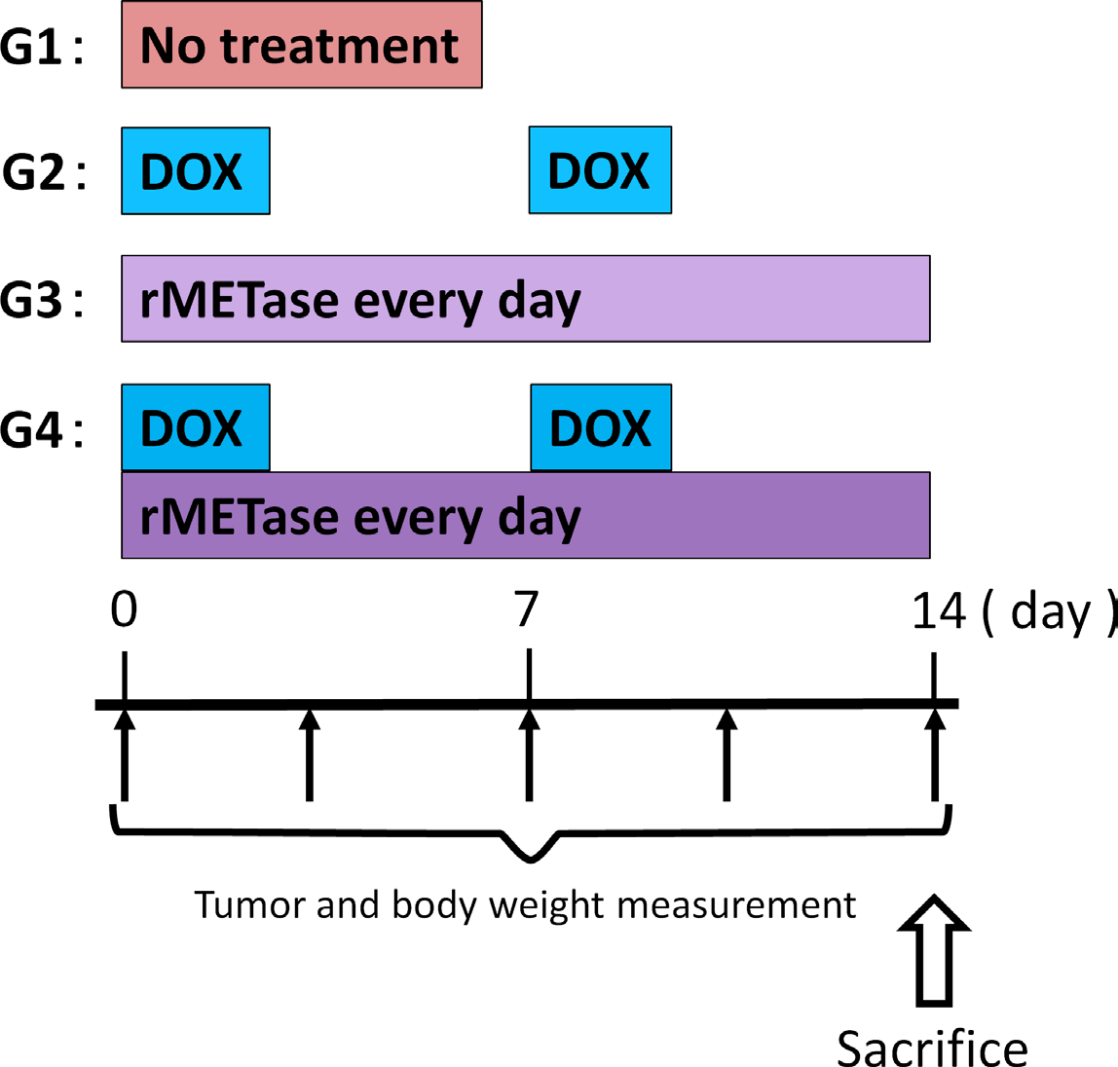
Treatment schema

**Figure 2:**
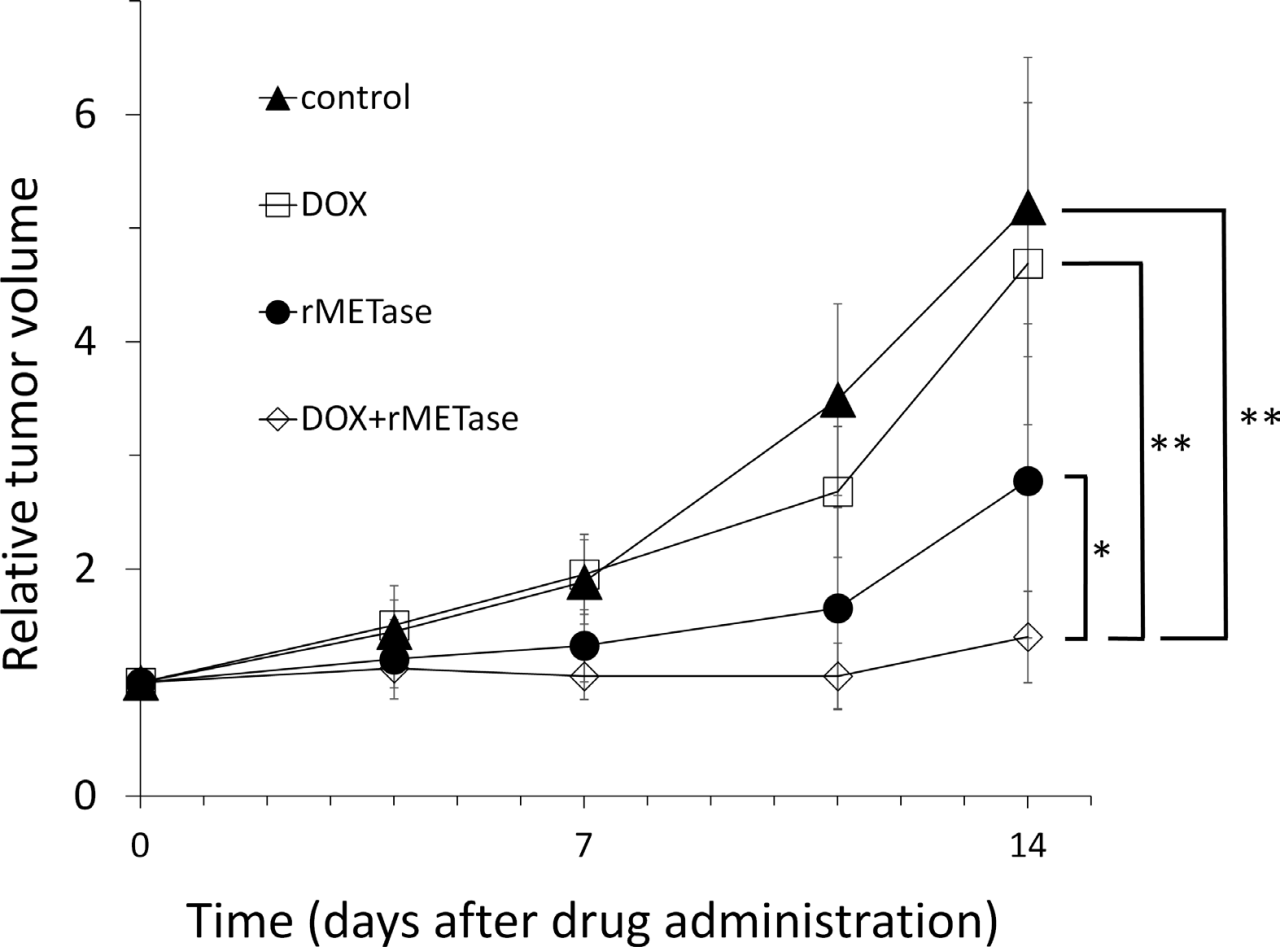
*In vivo* antitumor efficacy of doxorubicin (DOX), l-methionine α-deamino-γ-mercaptomethane lyase (rMETase) and DOX combined with rMETase Synovial sarcoma was grown orthotopically in the right biceps femoris muscle of nude mice and allowed to form tumors. Treatment, dose, route, and schedule were: DOX (3 mg/kg/week i.p. for 2 weeks), and rMETase (100 U/mouse/day, i.p., for 14 days). Relative tumor volume, shown by the line graphs, is the tumor volume at the indicated time points during the time of treatment divided by the tumor volume at the onset of treatment. *N* = 8 mice/group. ^*^p < 0.05, ^**^p < 0.001.

**Figure 3:**
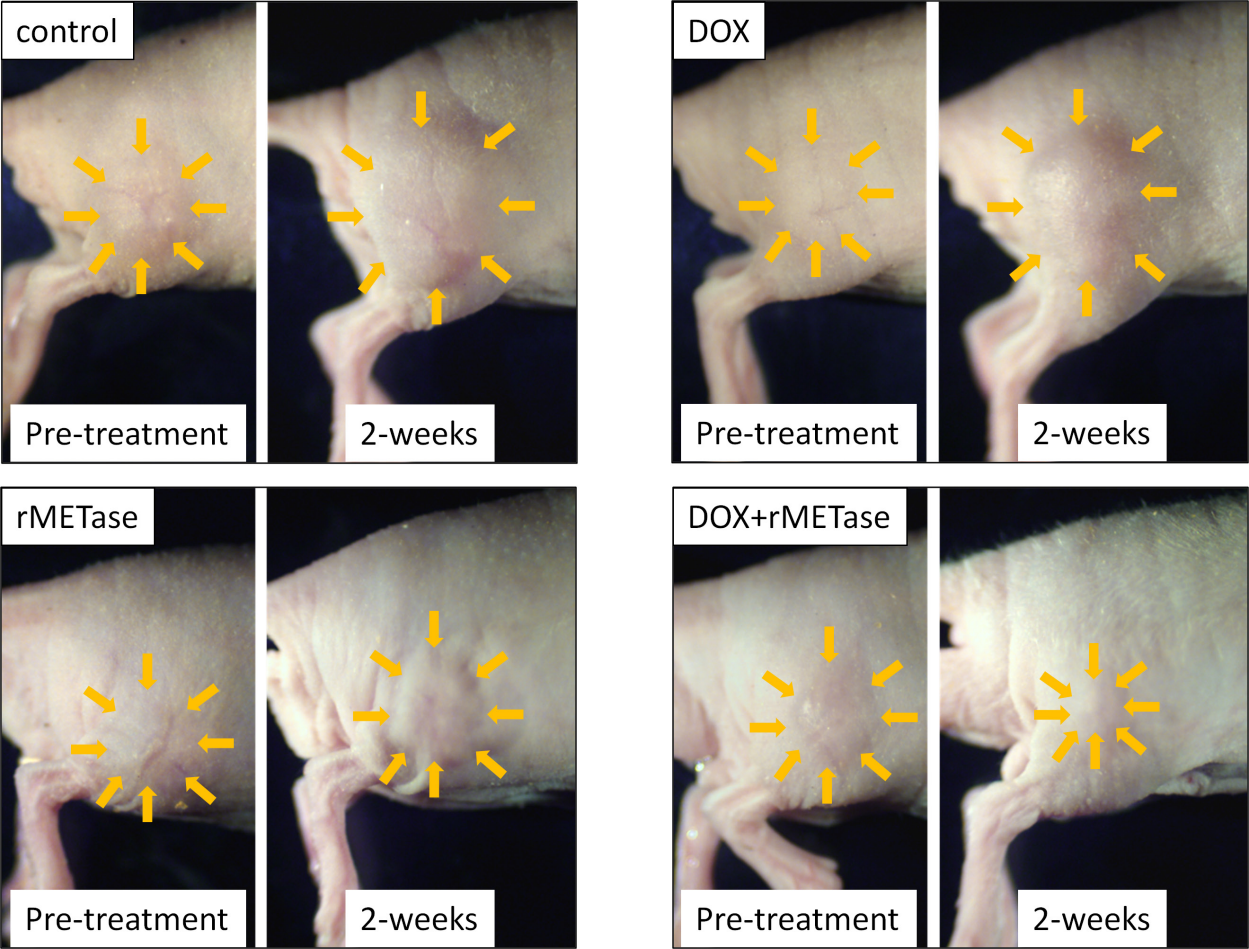
Photos of representative treated and untreated SS PDOX tumor

**Figure 4:**
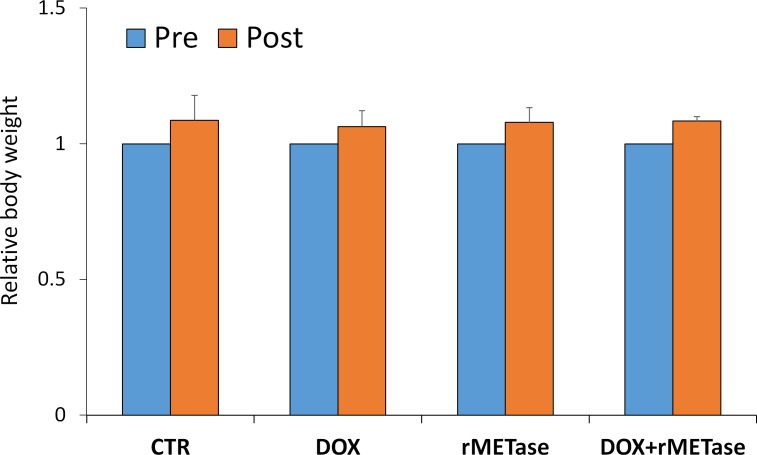
Bar graph shows body weight in each group at pre-treatment and 2 weeks after drug administration There were no significant differences between any group.

On day 14 after initiation, DOX (*p =* 0.48) was ineffective on the SS PDOX. rMETase (*p <* 0.005) inhibited tumor growth and DOX combined with rMETase (*p <* 0.0001) regressed tumor growth. DOX combined with rMETase was significantly more effective than both DOX alone (*p <* 0.001) and rMETase alone (*p <* 0.05) (Figures 1–3). The relative body weight on day 14 compared with day 0 did not significantly differ between any treatment group (Figure 4).

### Histology

**Figure 5:**
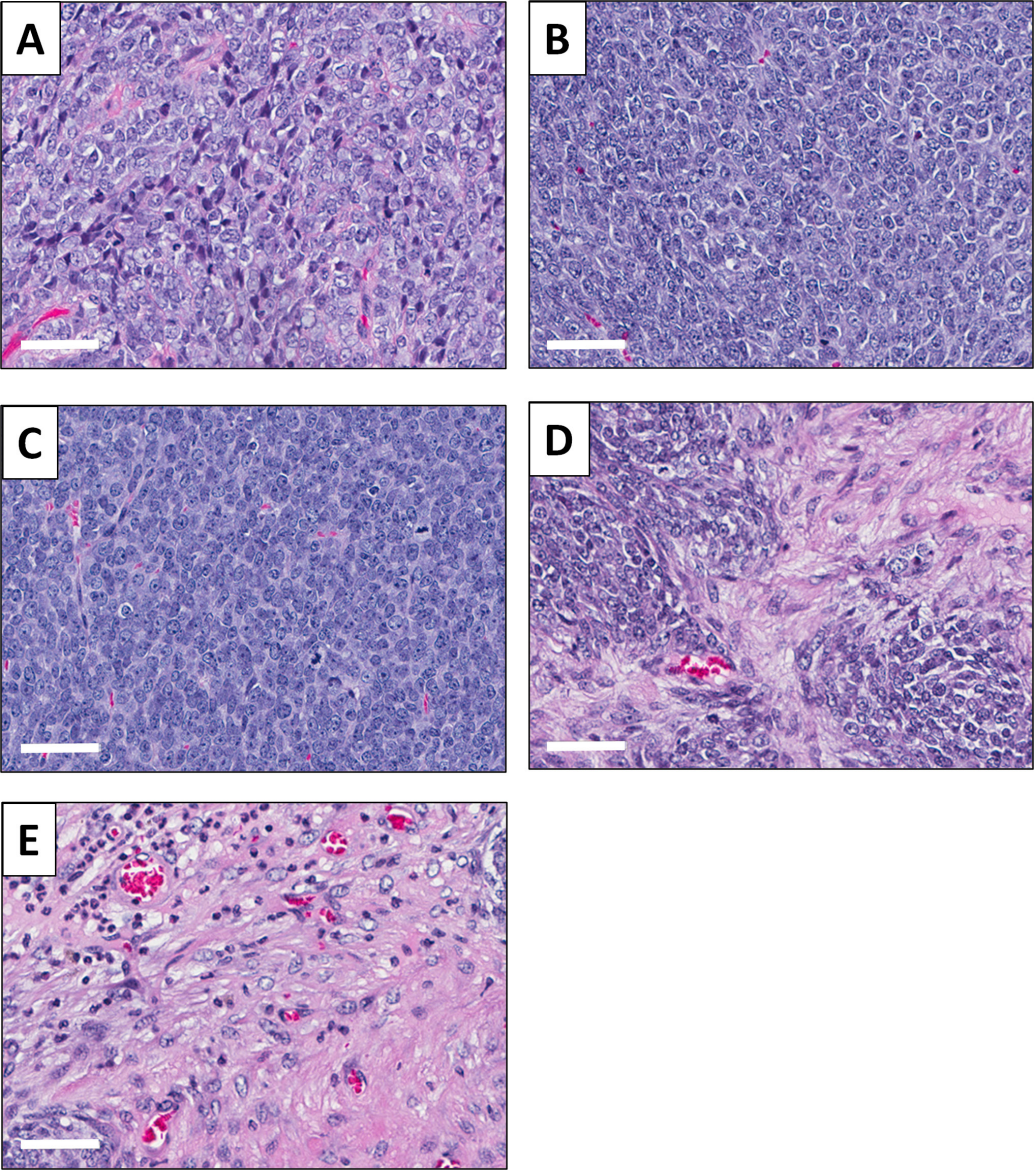
Tumor histology (**A**) H&E-stained section of the original SS patient tumor; (**B**) untreated SS PDOX tumor; (**C**) SS PDOX tumor treated with DOX; (**D**) SS PDOX tumor treated with rMETase and (**E**) SS PDOX tumor treated with DOX combined with rMETase. White scale bars: 50 µm.

High-power photomicrographs of hemotoxylin and eosin (H&E)-stained sections of the original patient tumor showed cancer cells with hyperchromatic, enlarged nuclei. Mitotic figures and atypical cells are present (Figure 5A). A high-power view of the untreated SS PDOX model showed similar features, including cells with hyperchromatic and enlarged nuclei. Mitotic figures, including atypical forms, are also present (Figure 5B). Tumors treated with DOX comprised viable cells without apparent necrosis or inflammatory changes (Figure 5C). Tumors treated with rMETase show altered cancer-cell shape and some necrosis (Figure 5D). Tumors treated with DOX combined with rMETase have extensive necrosis (Figure 5E).

There was a statistically-significant difference in tumor growth between either the untreated control or the DOX-treated PDOX, on the one hand, and either the rMETase- or the combination of rMETase and DOX-treated PDOX on the other. There was also a statistically-significant difference between the rMETase-treated and the combination rMETase-treated and DOX-treated PDOX. During the treatment period, there was no body-weight loss in any treated group.

Methionine restriction of cancer results in an S/G_2_-phase cell-cycle arrest that eventually leads to cancer-cell death [29–31]. This is the probable mechanism for necrosis in the rMETase-treated PDOX which is further exacerbated by the addition of DOX to rMETase.

Lung metastasis in the synovial sarcoma and its sensitivity to rMETase will be a topic for future study.

Our laboratory has developed PDOX mouse models of cancer for discovery of transformative therapy for recalcitrant cancer. The PDOX nude mouse model is established with the technique of surgical orthotopic implantation (SOI). These models include breast [32], ovarian [33], lung [34], cervical [35], colon [36–38], pancreatic [39–43] and stomach cancer [44], melanoma [21, 22, 24, 45–48] as well as sarcoma [2–20, 23, 25, 26]. The PDOX model, developed by our laboratory over the past 30 years, has many advantages over subcutaneous-transplant models which grow ectopically under the skin and rarely can metastasize [49].

We have recently demonstrated that rMETase is effective against a PDOX model of Ewing’s sarcoma [7]. We have also found rMETase to be effective in a PDOX model of DOX-resistant spindle cell sarcoma [26] and to overcome DOX-resistance in the spindle-cell sarcoma PDOX [23].

All PDOX models tested thus far, including those derived from Ewing’s sarcoma [7], osteosarcoma [15], spindle-cell sarcoma [23, 26], and melanoma [21, 22, 24] are very sensitive to rMETase, and in combination with first-line chemotherapy, rMETase can regress PDOX tumors. These results, in addition to extensive *in vitro* and *in vivo* cell line studies showing MET-dependence and response to rMETase or other means of MET-restriction [29, 30, 50–55] suggest rMETase is a general therapeutic for all cancer. Extensive safety tests were performed with rMETase in primates [56, 57] and initial testing in humans [58, 59] indicated minimal toxicity of rMETase. Very recently we have shown that rMETase can be administered orally [24], suggesting the near-future widespread use of rMETase for all types of cancer in the clinic.

Methionine dependence is due to the overuse of methionine for aberrant transmethylation reactions in cancer and is possibly the only known general metabolic defect in cancer [50, 60–66].

The overuse of methionine by cancer cells for enhanced and unbalanced transmethylation may be the basis of the methionine dependence of cancer cells and is termed the “Hoffman effect”, analogous to the Warburg effect of glucose overuse in cancer [64]. The Hoffman effect can be observed clinically in [^11^C]MET PET imaging which gives a much stronger signal than fluorodeoxyglucose (FDG)-PET [67].

Previously-developed concepts and strategies of highly-selective tumor targeting can take advantage of molecular targeting of tumors, including tissue-selective therapy which focuses on unique differences between normal and tumor tissues [68–73].

## MATERIALS AND METHODS

### Mice

Athymic *nu/nu* male nude mice (AntiCancer, Inc., San Diego, CA), 4–6 weeks old, were used in this study. All mice were kept in a barrier facility on a high efficiency particulate arrestance (HEPA)-filtered rack under standard conditions of 12-hour light/dark cycles. The animals were fed an autoclaved laboratory rodent diet. All animal experiments were performed with an AntiCancer Institutional Animal Care and Use Committee (IACUC)-protocol specifically approved for this study and in accordance with the principals and procedures outlined in the National Institutes of Health Guide for the Care and Use of Animals under Assurance Number A3873-1. Anesthesia and analgesics were used for all surgical experiments to avoid excessive suffering of the mice. A ketamine mixture (0.02 ml solution of 20 mg/kg ketamine, 15.2 mg/kg xylazine, and 0.48 mg/kg acepromazine maleate) was used subcutaneously for all mice. The animals were monitored carefully during surgery to keep adequate depth of anesthesia. The animals were observed daily and humanely sacrificed by CO_2_ inhalation when they met the following criteria: severe tumor burden (more than 20 mm in diameter), prostration, significant body weight loss, difficulty breathing, rotational motion and body temperature drop.

### Patient-derived tumor

A 45-year-old male diagnosed with primary synovial sarcoma on the lower leg underwent surgical resection at the Department of Surgery, University of California, Los Angeles (UCLA). Written informed consent was obtained from the patient as part of a UCLA Institutional Review Board (IRB #10-001857)-approved protocol. The patient received neo-adjuvant doxorubicin-based chemotherapy prior to surgery.

### Surgical orthotopic implantation (SOI) for establishment of PDOX model

A fresh sample of the tumor of the patient was obtained and transported immediately to the laboratory at AntiCancer, Inc., on wet ice. The sample was cut into 5 mm fragments and initially implanted subcutaneously in nude mice. The grown tumors were cut into small fragments (3–4 mm). After nude mice were anesthetized, a 5 mm skin incision was made on the right high thigh, and biceps femoris was split to make space for the tumor. A single tumor fragment was implanted orthotopically into the space to establish a PDOX model. The wound was closed with 6-0 nylon suture (Ethilon, Ethicon, Inc., NJ, USA).

### rMETase production

The pAC-1 rMETase high-expression clone was used for rMETase production. The fermentation procedure for host *E.coli* cells and the purification protocol for rMETase were the same as previously described: rMETase was purified by 3 different steps using columns of DEAE Sepharose FF, Sephacryl S-200HR, and ActiClean Etox, which is designed for eliminating endotoxin [74].

### Treatment study design in the PDOX model of synovial sarcoma

SS PDOX mouse models were randomized into 4 groups of 8 mice each: G1, control without treatment; G2, Doxorubicin (DOX) (3 mg/kg, intraperitoneal [i.p.] injection, weekly, for 2 weeks); G3, rMETase (100 unit/mouse, i.p., daily, for 2 weeks); G4, DOX (3 mg/kg), i.p. weekly, for 2 weeks) combined with rMETase (100 unit/mouse, i.p., daily, for 2 weeks) (Figure 1). Tumor length, width and mouse body weight were measured twice in a week. Tumor volume was calculated with the following formula: Tumor volume (mm^3^) = length (mm) × width (mm) × width (mm) × 1/2. Data are presented as mean ± SD.

### Histological examination

Fresh tumor samples were fixed in 10% formalin and embedded in paraffin before sectioning and staining. Tissue sections (3 μm) were deparaffinized in xylene and rehydrated in an ethanol series. Hematoxylin and eosin (H&E) staining was performed according to standard protocol. Histological examination was performed with a BHS system microscope. Images were acquired with INFINITY ANALYZE software (Lumenera Corporation, Ottawa, Canada).
